# Longitudinal stability of gel T1 MRI Phantoms for quality assurance of T1 mapping

**DOI:** 10.1186/1532-429X-17-S1-W28

**Published:** 2015-02-03

**Authors:** Vassilis Vassiliou, Ee Ling Heng, Jackie Donovan, Andreas Greiser, Sonya V Babu-Narayan, Michael A Gatzoulis, David Firmin, Dudley J Pennell, Peter Gatehouse, Sanjay K Prasad

**Affiliations:** 1 CMR, Royal Brompton Hospital, London, UK; 2 Department of Biochemistry, Royal Brompton Hospital, London, UK; 3 National Heart and Lung Institute, Imperial College London, London, UK; 4 Siemens, Germany, Germany

## Background

Quantification of interstitial (diffuse) myocardial fibrosis by T1 mapping may prove useful in a range of conditions. Both native T1 maps and extracellular volume fraction (ECV) are being increasingly used clinically and have been shown to be sensitive to a wide range of conditions and associate with worse prognosis. However, quality control by T1 phantoms is essential [1,2] and their long-term stability has not been demonstrated. Phantoms with long term stability could assure the stability of methods applied to patients against scanner alternations and across multiple centers. We sought to investigate the long-term stability of phantoms prepared to model T1 and T2 of blood and myocardium over a period of 52 weeks.

## Methods

NiCl_2_-agarose gel phantoms [3] were prepared in a standardised lab following a reproducible procedure to model T1 and T2 of blood and myocardium, native and post-contrast [4]. Phantoms were 60ml glass narrow-neck sealed thick-wall bottles, filled with minimal gaps from gel contraction whilst cooling. These were kept in a temperature controlled MRI room and imaged weekly for 52 consecutive weeks (Siemens, Avanto, 1.5T) using consistent coil and phantom arrangement with a 11-RR MOLLI prototype (Siemens WIP448B) with high resolution (for heart rate 75bpm) and low resolution (for heart rate 100bpm) versions, with pre-contrast and post-contrast variants (4) plus spin-echo T2. Image parameters were identical weekly except for automatic adjustments of flip-angle and reference frequency. T1 and T2 values were taken as mean values in each phantom in pixel-wise maps.

## Results

**Figure 1:**
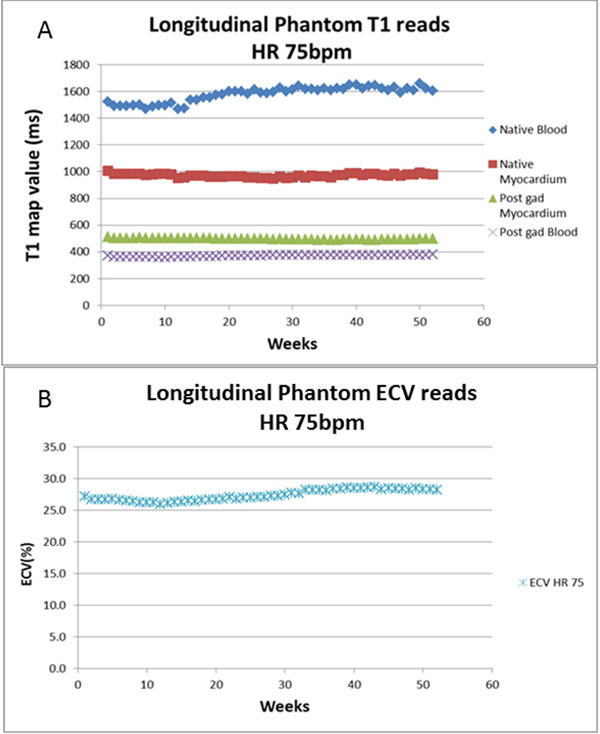
showing longitudinal trend for T1 maps and ECV. All T1 parameters remained relatively stable, with native myocardium remaining the most stable. This is particularly useful for T1 mapping sequences only utilizing native myocardial values. The ECV also remained relatively stable during these 52 weeks, suggesting that gel phantoms can be accurately used in the quality assurance of T1 mapping.

Both high resolution and low resolution sequences had similar T1 means, standard deviation and coefficient of variation (CoV) for blood and myocardium native and post-gadolinium (Table 1). CoV was higher for the longer T1 and T2 values. Specifically for the T1 values, there was no significant drift seen in the shorter T1 values (native myocardium and post-gad blood and myocardium) but there was an increase in the longer native blood value of over one year in accordance to the following equation: T1 native blood= 3.25 x no of weeks + 1492, suggesting an average increase of 169ms over the year (Figure 1). Native blood T1 was the most stable parameter following: T1 native myocardium= -0.01 x no of weeks + 972 suggesting a decrease of 1ms over this period.

**Table 1:** showing the variability during a 52 week period of the Phantom T1 and T2 values and ECV using an 11-cycle 8-image prototype MOLLI. There were 2 phantoms for each T1 and T2 value prepared in standardised laboratory and the value used here represents the average of the two. The phantom ECV was calculated using a hematocrit of 0.43.

	T1						T2		
	**Mean (ms)**	**SD**	**CoV (%)**	**Mean (ms)**	**SD**	**CoV (%)**	**Mean (ms)**	**SD**	**CoV (%)**

	High Resolution (75bpm)			Low resolution (100bpm)			Spin-echo protocol		

**Native Myocardium**	972	12.6	1.3	972	13.6	1.4	56	1.3	2.2

**Native Blood**	1579	56.4	4.6	1550	61.9	4.0	234	8.0	3.4

**Post Gd Myocardium**	498	5.1	1.0	497	13.5	1.4	48	0.9	1.9

**Post Gd Blood**	372	6.4	1.7	372	6.5	1.8	149	3.0	2.0

**ECV**	27.4%	0.9	3.2	27.6%	0.9	3.1			

## Conclusions

There was significant stability of the NiCl_2_-agarose gel phantoms over a one year period, justifying their routine use in clinical practice to support T1 mapping. Having determined stability, detection of parameters that could lead to phantom value inaccuracies (e.g. heart rate, flip angle and temperature variation) may require further investigation.

## Funding

NIHR Cardiovascular Biomedical Research Unit of Royal Brompton & Harefield NHS Foundation Trust and Imperial College London.
